# Flax fibre reinforced alginate poloxamer hydrogel: assessment of mechanical and 4D printing potential†

**DOI:** 10.1039/d4sm00135d

**Published:** 2024-05-02

**Authors:** Charles de Kergariou, Graham J. Day, Adam W. Perriman, James P. K. Armstrong, Fabrizio Scarpa

**Affiliations:** a Bristol Composites Institute, School of Civil, Aerospace and Design Engineering (CADE), University of Bristol, University Walk Bristol BS8 1TR UK hl18503@bristol.ac.uk charles.dekergariou@bristol.ac.uk; b Biomedical Engineering, James Watt School of Engineering, University of Glasgow Glasgow UK; c School of Cellular and Molecular Medicine, University of Bristol BS8 1TD Bristol UK; d Research School of Chemistry and John Curtin School of Medical Research, Australian National University Canberra ACT2601 Australia; e Department of Translational Health Sciences, Bristol Medical School, University of Bristol BS1 3NY Bristol UK

## Abstract

The mechanical and printing performance of a new biomaterial, flax fibre-reinforced alginate-poloxamer based hydrogel, for load-bearing and 4D printing biomedical applications is described in this study. The-self suspendable ability of the material was evaluated by optimising the printing parameters and conducting a collapse test. 1% of the flax fibre weight fraction was sufficient to obtain an optimum hydrogel composite from a mechanical perspective. The collapse test showed that the addition of flax fibres allowed a consistent print without support over longer distances (8 and 10 mm) than the unreinforced hydrogel. The addition of 1% of flax fibres increased the viscosity by 39% and 129% at strain rates of 1 rad s^−1^ and 5 rad s^−1^, respectively, compared to the unreinforced hydrogel. The distributions of fibre size and orientation inside the material were also evaluated to identify the internal morphology of the material. The difference of coefficients of moisture expansion between the printing direction (1.29 × 10^−1^) and the transverse direction (6.03 × 10^−1^) showed potential for hygromorphic actuation in 4D printing. The actuation authority was demonstrated by printing a [0°; 90°] stacking sequence and rosette-like structures, which were then actuated using humidity gradients. Adding fibres to the hydrogel improved the repeatability of the actuation, while lowering the actuation authority from 0.11 mm^−1^ to 0.08 mm^−1^. Overall, this study highlighted the structural and actuation-related benefits of adding flax fibres to hydrogels.

## Abstract 2

A new 3D bioprinted biomaterial, with flax fiber-reinforced alginate hydrogel, is developed. The study assesses the impact of flax fibers on 3D printing, mechanical, 4D printing, and geometric properties of the composite.

## Graphical abstract

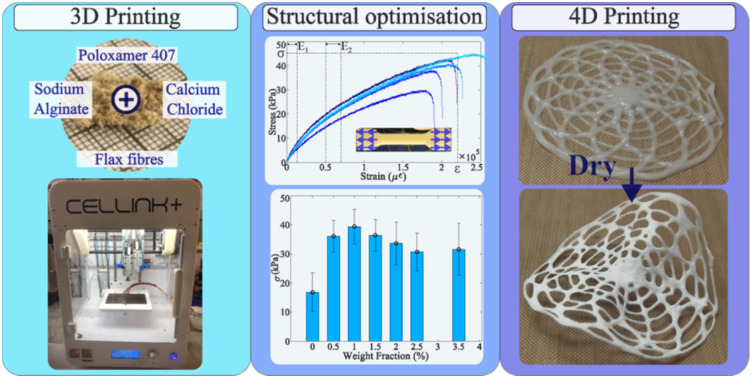

## 1 Introduction

Hydrogels are extensively used in many biomedical applications, such as 3D cell cultures,^1,2^ wound healing^3^ and bone repair.^4^ These can be natural (*e.g.* collagen, fibrin, hyaluronic acid (HA), alginate), synthetic (polyvinyl alcohol (PVA), (polyethylene glycol (PEG))), poly(*N*-isopropylacrylamide) (PNIPAm) or semi-synthetic (HA–PEG).^5^ Natural hydrogels based on alginate are one of the most widespread biomaterial used in tissue engineering.^6^ However, alginate-based solutions are complex to print due to their low viscosity.^6^ Hence, alginate can be combined with the surfactant poloxamer, for instance, as wound dressing materials^7^ or for 3D bioprinting.^8^ However, the range of accessible mechanical properties in hydrogels is somewhat limited, and precludes their more widespread adoption in materials science.

**Fig. 1:**
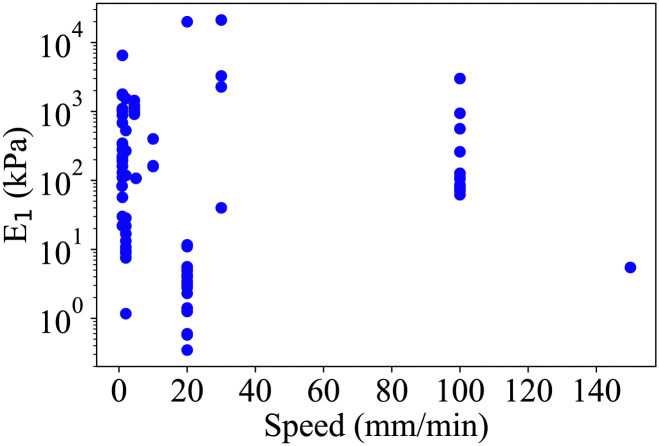
Open literature data related to loading speed and Young's modulus (*E*_1_) of alginate-based hydrogels.^16–37^

Techniques such as the addition of tannic acid and Fe^3+^ ions to strengthen and toughen alginate hydrogels were previously developed.^9^ The addition of fibre was observed to have the same potential.^10^ Alginate/poloxamer gels could also be used as multifunctional platforms to carry electric currents.^11^ Particles can be added to these hydrogels for changing their mechanical and biomedical properties.^12,13^ When added to hydrogels, flax fibres have shown cytocompatibility and biosafety.^14^ These fibres were implemented as a net in PVA hydrogel to highlight their greater mechanical properties, water absorption capability but lower thermal stability compared to polypropylene and jute reinforced PVA hydrogels.^15^ However, to the authors' knowledge, flax fibres have not been evaluated to reinforce alginate-based hydrogels. Fig. 1 features the stiffness and corresponding tensile loading rates for alginate hydrogels found in open literature. The papers from which these data were extracted were selected as papers answering to the following web of science search: (all fields: 3d print* AND All fields: alginate AND all fields: hydrogel AND All fields: tensile).

From Fig. 1, it is clear that there is a significant variation in stiffness among the currently available 3D-printed alginate hydrogels. This variation can be attributed to differences in loading rates, specimen shapes, hydrogel constituents-(poloxamer,^38^ sodium alginate^39^), and production processes. For instance, the addition of poloxamer into alginate-based hydrogel was shown to improve the compression stiffness of the material.^8^ Crosslinking strategies also introduce variability in the stiffness of the hydrogel. For instance, Bari *et al.* used a mixture of a 2% calcium chloride and 5% protamine solution^16^ to cross link their hydrogel for 1 hour. On the other hand, Gharai *et al.* immersed the hydrogel studied in a 4% CaCl_2_ solution for 30 minutes.^26^ The wide range of results reflects the diverse nature of these factors. For instance, Kaliampakou *et al.* have optimised the printing conditions of 3D printed hydrogels to obtain the optimal printing precision.^40^ With such a large scatter of mechanical properties due to the diversity of the influencing parameters, researchers would benefit from improved modelling of the material. For instance, representative volume element models are used to forecast the macroscopic properties of a composite material consisting of a hydrogel and its microscale reinforcement. The range of strain considered for the measurement of the Young's modulus also varies significantly within the different studies considered. Furthermore, it is important to note that the measurements of Young's modulus often provide a representation of stiffness within a limited range of material deformations. To obtain a more comprehensive understanding of the hydrogel's behaviour over a wider range of strains, it becomes necessary to employ appropriate material models. Several models have been developed to characterize the mechanical properties of hydrogels, including the Neo-Hookean, Mooney-Rivlin, Yeoh, and Biderman models. These models enable a more accurate and comprehensive characterisation of the mechanical response of hydrogels, enhancing our understanding of their behaviour under various loading conditions.^41^ The characterisation of the materials using these mechanical models also allows a better quantitative appreciation of the variability of the properties highlighted in Fig. 1.

Several papers have highlighted the potential deformation and actuation potential of hydrogel systems.^42,43^ Gelatin fibres have provided 4D printing capabilities in hydrogels.^44^ Baker *et al.* have produced 4D printing multilayer origami-shaped structures from non-reinforced polyurethane-based hydrogels origami-shaped structures.^45^ Bakarich *et al.* created a thermally PNIPAAm-actuated hydrogel valve to open or close, depending on the temperature of the water.^46^ This class of hydrogel was remarkable for its robustness and polymer-type of behaviour, with an elastic and a plastic phase when loaded in tension. All these studies have shown interesting potential for hydrogels to be used as 4D printed actuators. However, few of them have displayed ways of improving the actuation capability of one material such as.^47^ Flax fibres have shown some significant potential as reinforcement in 4D printed fibre composites.^48,49^ However, they have never been combined with hydrogels to create actuation-capable structures.

The present work targets the following objectives, to understand the mechanical and actuation potential of the new material created:

• To develop a new composite material of alginate/poloxamer hydrogel reinforced by flax fibres.

• To evaluate the printability of the material, as well as the impact on the printability provided by the presence of the flax fibres.

• To evaluate the mechanical performance of the hydrogel/flax fibre composite material.

• To characterize the external and internal architecture of the composite material.

• To assess the ability of this material to construct 4D printed structures.

Several parameters were assessed to produce an optimised version of this hydrogel composite. The printability of the optimised material was assessed *via* collapse and fusion filament tests to assess its self-suspendable ability and resolution, respectively. 3D printing annulus scaffold was also conducted to demonstrate the repeatability of the constructs obtained with added flax fibres. The mechanical potential of this material was evaluated *via* tensile and shear using uniaxial tensile machines and a rheometer. The parameters of hyperelastic Mooney-Rivlin models were also obtained further to characterise the behaviour of the hydrogel without fibres. The annulus scaffold is a shape widely found in the human body. Hence, the constructs were tested in compression to provide the order of magnitude comparison with matter found in living beings. The mechanics of the flax fibres was analysed *via* single-fibre tensile testing to assess better the impact of the reinforcement on the hydrogel matrix. The internal architecture of the hydrogel/fibres composite was investigated *via* scanning electron microscopy, computed tomography (CT) scanner and optical microscopy. Those topological data provided the information to generate a representative volume element of the composite material. Finally, the potential for this fibre-reinforced hydrogel composite in 4D printing was determined by measuring the ability of the material to expand and contract deferentially in various directions. Finally, a rose window-shaped construct was 4D printed to highlight the actuation potential of the material.

## 2 Methods and production

### 2.1 Flax fibre production

Continuous flax fibres from ecothechnilin were manually cut to approximately 1 cm long and then placed in a planetary ball mill. The fibres were milled for 15 min with 30 mm diameter balls at 650 rpm with a minute pause for every minute of milling. The fibres were then milled for 15 min at 650 rpm with 10 mm balls, pausing three minutes every minute of the operation. With this procedure, the resulting fibres had sufficiently small dimensions for extrusion with a 3D printer.

### 2.2 Hydrogel production

**Fig. 2:**
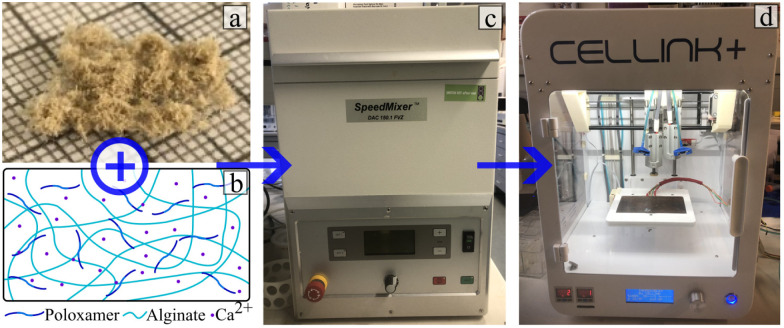
Production of the composite hydrogel. (a) Flax fibres (b) hydrogel chemicals (from left to right: sodium alginate; poloxamer 407; calcium chloride dihydrate). (c) SpeedMixer DAC 150.1 FVZ centrifuge used to mix the hydrogel and the flax fibres. (d) Cellink+ bio printer used to prepare the composite.

The composite hydrogel was produced by mixing the different components presented in Table 1. The poloxamer and sodium alginate were purchased from Sigma-Aldrich with a viscosity of 5–40 cps at 1% in water at 25 °C and a quality level of 400. The poloxamer 407 had a quality level of 100. The details of the hydrogel production procedure are given in Table S1 in ESI.† As shown in Fig. 2, the specimens for the different tests were printed with an Inkredible + ^*TM*^ Cellink printer with a nozzle (length: 6.35 mm; diameter: 0.838 mm) kept at 37 °C. The speed of the printing head during extrusion was constant at 240 mm min^−1^. The specimen was cross-linked in a 10 mM solution of calcium chloride for 24 h before the different tests were performed. The extrusion pressures are 40, 75, 75, 70, 70, 80 and 85 kPa for flax fibre weight fraction 0%, 5%, 10%, 15%, 25% and 35%, respectively.

**Table 1:** Weight proportion of material in the hydrogel

Material	Water	Sodium alginate	Poloxamer	Flax fibre	Calcium chloride
Ratio	69.8%	6.0%	13.2%	1.0%	10.0% (200 mM)

### 2.3 Single fibre tests

Flax fibres with 40 mm gauge length were glued to end tabs with Loctite super glue on each side. After 24 hours of glue drying, the five fibres were inserted into the uncross-linked hydrogel. The hydrogel was then cross-linked with 10 mM calcium chloride for 24 h. The six dried and five wet fibres had their diameters measured *via* microscope inspection (Axioscope Zeiss). Each fibre had five images taken along its gauge length to measure two different diameter values on each image. A Dia-Stron LEX820 extensometer machine (Dia-Stron Limited, Andover, UK) with a 20 N load cell and a strain rate of 0.02 mm s^−1^ was then used to run the single fibre tests following the protocol suggested by Kandemir *et al.*^50^

### 2.4 Fibre dimensions

The fibres were crushed and therefore, a myriad of fibres sizes were obtained and were used to reinforce the hydrogel. The dimensions of the fibre were quantified to understand their impact on the properties of the reinforced hydrogel. First, flax fibres were selected using the same method as for the production of flax fibre-reinforced hydrogels. Deionised water was added inside a Petri dish containing fibres, which was then positioned in a FASEP machine (IDM systems). The machine was calibrated to measure the width, aspect ratio and length of all fibres with a length larger than 50 μm.

An image data processing technique was also used to measure the dimensions of the flax fibres. A Petri dish containing fibres and deionized water was placed under the microscope, and ten polarized images were captured using a 10× objective lens. These images were then converted to grayscale with the python function convert (“L”) to enable differentiation between the fibres and the background. The centroid position for each block of black pixels, corresponding to the fibres, was calculated to determine the dimensions. The length of the fibre represented by this block of pixels was determined as twice the distance between the centroid, and the pixel furthest away from the centroid. The width of the fibre was measured from the intersection between the contour of the block and the perpendicular straight line to the length of the fibre. These measurements of the width, length and determination of the aspect ratio were repeated for all the black blocks. To combine the measurements obtained using the FASEP and the microscope-based techniques, a common interval of measurement sizes between 50 μm and 100 μm was selected. The number of fibres measured using the microscope-based approach was then increased to match the number of fibres falling within this common range. The numbers of fibres measured using the two techniques were finally assembled and histogram distributions were obtained. The distributions of the length widths and aspect ratio were then interpolated using eqn (1) with (*a*, *b*, *c*, *d*, *e*, *f*, *g*, *h*, *k*) ∈ *R*^9^.

1 *f*(*x*) = *a* × *e*^−*b*×*x*^ + *c* × *e*^*d*×*x*^ + *e* × *x*^3^ + *f* × *x*^2^ + *g* × *x* + *h* + *k*/*x*

The interpolation functions for the length and the width were obtained using the minimize function from the scipy module in python. The interpolation function for the aspect ratio was obtained using the fit function of Matlab. A three-parameter distribution can provide a comprehensive characterisation of the fibres within the hydrogel. The distribution must however fit the relation presented in eqn (2). In this eqn (1), ar and *w* represent length, aspect ratio and width, respectively.

2 *l* = *ar* × *w*

To generate a list of fibre dimensions that adhered to these constraints, approximately 400 000 data points were used, ensuring compliance with the previously measured distribution. Permutations of the aspect ratio data points were then performed to obtain the optimal length distribution. This was achieved by calculating the product of each aspect ratio and width term. A genetic algorithm was then used to obtain the optimal permutation of the aspect ratio data points. The objective function of the optimisation was the minimisation of the Euclidean norm between the values of the length obtained *via* the algorithm and the ones extracted from the tests.

### 2.5 Tensile test

The specimens were subjected to tensile loading using a Starrett FMS500 machine with a 10 N load cell and a loading speed of 1 mm min^−1^, as suggested by the ASTM D3039 standard for composite materials. The dog bone shape and the dimensions of the specimens printed for the tests are shown in Fig. S1 in ESI.† A minimum of five specimens were used for each conducted test. The precise dimensions of the specimens were measured by capturing photos and analyzing them using ImageJ Fiji software. The properties obtained from the tensile tests were measured by considering smoothed load data that was averaged over seven data points over 0.001 mm displacement range.

Across all fibre content variations, the stress–strain curves exhibit a distinct inflexion point (see Fig. S2 in ESI†), effectively dividing each curve into two distinct regions. A modulus is therefore defined on each side of the inflexion point: *E*_1_ between 0 *με* and 10 000 *με* and *E*_2_ between 50 000 *με* and 70 000 *με*. The maximum load point was considered to determine the strength (*σ*) and the value of the strain at failure (*ε*). The point of maximum load is also used as a limit to calculate the area under the curve and therefore a representation of the energy dissipated during the fracture of the specimen. KrusKal Wallis test were conducted to observe the impact of the amount of flax fibre added on the stiffness of the composite.

#### 2.5.1 CT scanning

One tensile test specimen was inspected using a CT scanner (Nikon XTH320 with a 50 kV voltage, scanning current of 250 μA). The resolution of the run was 6 μm over 2000 images. The CT scanner was used to obtain a conservative estimate of the porosity of the hydrogel based on voids larger than 6 μm.^51^ The stack of images obtained from the scan was divided into five different sections along the length of the specimen.

#### 2.5.2 Hydrogel properties

A two-parameter Mooney Rivlin model was used to interpolate the mechanical behaviour of the hydrogel.^52^ Stress–strain data from the tensile tests were uploaded to the ANSYS Multiphysics code to identify the *C*10 and *C*01 coefficients characteristics of this model.

### 2.6 Collapse test

A raise 3D pro plus printer was used to produce the poly(lactic acid) support for the collapse tests. Initial trials were performed to define the gap distances of the support. The final dimensions of the support for the collapse test are 4 mm, 5 mm, 6 mm, 8 mm and 10 mm. The support has similar geometry than the one used by previous studies^53–55^ The material was printed on the edge of the support with an EPL zoom webcam 2MPixel camera positioned on the side of the printer to record the procedure. The footage from the camera was utilized to determine the time at which the specimen failed. The starting point for the time measurement corresponded to the moment when the printer nozzle was positioned on top of the initial edge of the support. After the printing process was completed, approximately 150 s were allowed to elapse to observe if any material failure occurred. For each printing parameter tested, ten specimens were used.

### 2.7 Fusion filament test

Five specimens were prepared with 1% and without flax fibres. The distance between filaments ranges from 0.5 mm to 2.1 mm with a 0.1 mm increase increment, as shown in Fig. S3 in ESI.† Immediately after the print, a photo on top of the printed structure was taken. The distance between filaments, the width of the filament and the width of the corners were measured from this photo in Matlab.

### 2.8 Annulus scaffold

Fig. S4 in ESI,† shows the printing path used to print the ten annulus scaffold used as specimens. The annulus scaffold was crosslinked before compression and dimensions measurement. The compression tests were performed using a Starrett FMS500 machine with a 10 N load cell and a loading speed of 1 mm min^−1^, until 8 N were reached. The modulus was measured between 1000 μm and 3000 μm, as suggested by the ASTM D6641 standard and shown in Fig. S5 in ESI.†

### 2.9 Rheology test

The specimens, dimensions and the G-code used for the test are presented in Fig. S6 in ESI.† The rheometry tests were carried out at 1 Hz as suggested by the developer of the equipment (Discovery Rheometer HR2) to test collagen^56^ and alginate-based hydrogels.^57–59^ The frequency used also allowed the characterisation of the hydrogel under quasi-static conditions. The test was carried out at 25 °C in a strain sweep mode ranging from 10^−2^% to 10^1^% as shown *via* the stress strain curves in Fig. S7 in ESI.† The stiffening coefficient (SC) was calculated from eqn (3), with *μ*_Fibre_ being the viscosity of the reinforced material, *μ*_NoFibre_ the viscosity of the not reinforced material and *w*_Fibre_ the fibre weight fraction of the reinforcement:3
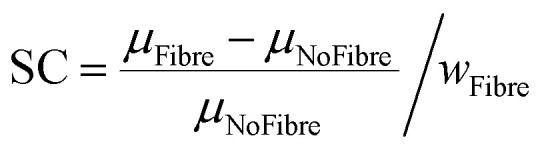


### 2.10 Orientation of the fibres

Although the fibres were generally aligned along the printing direction due to the shearing in the nozzle during the extrusion, they exhibited some discontinuity in terms of distribution. To estimate this spatial distribution, a tensile test specimen was cut along the width and thickness directions in a bevel with a sharp blade. The surfaces were then positioned in a high vacuum SEM to acquire back-scattered electron images with a 15 kV electron beam. A 100× magnification was considered sufficient to obtain the images and allowed the identification of 200 fibres for the measurement. In the case of perfectly cylindrical fibres, if a fibre is cut at a bevel, the largest dimension observed from the top view represents the in-plane orientation of the fibre itself. The flax fibres are not perfectly cylindrical, hence it was not always clear if the largest dimension was due to the orientation of the fibre, or to the shape of the flax fibre itself. As a result, a significant portion of the fibres was disregarded in this measurement. Additionally, an assumption of symmetry was made for the two measuring angles to determine the final distributions of the fibres.

### 2.11 Density and coefficient of moisture expansion

The density and the coefficient of moisture expansion were measured for 0% and 1% fibre weight fractions. At least five specimens with 20 mm × 20 mm × 10 layers (printing path and geometry can be found in Fig. S8 in ESI†) were produced for each type of composite material. The dimensions of the specimens (length *l*, width *w*; thickness *t*) and the mass *m* of the specimens were measured wet, immediately after cross-linking. The length, width and thickness correspond to the *x*, *y* and *z* directions in Fig. S8 (ESI†).

The dimensions of the material in the wet state were measured by taking photos of the top and side views of the specimen and measuring five times the associated dimensions with ImageJ Fiji. The mass was measured with a 0.001 g resolution Ohaus Adventurer^TM^ Precision balance. The density (*ρ*) and coefficient of moisture expansion (CME) were calculated with eqn (4) and (5) (with *d* ∈ {*l*, *t*, *w*}):

4 *ρ* = *m*/(*l* × *d* × *w*)

5
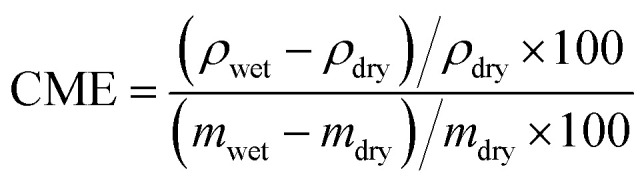


After performing those measurements, the specimens were dried at room temperature and humidity before measuring their dimensions and mass again (see Fig. S8(c), ESI†). The measurements in the dried state were performed when the mass of the specimens converged to a stable value after 72 h in excess. The volume of the specimens was measured in their dried state using the *EX*_*s*_*can-SP* 3D scanner from Shining 3D. An example of the volume (*v*) obtained from this scan is given in Fig. S8(d) (ESI†). The density of the dry specimens was calculated from eqn (6).

6 *ρ* = *m*/*v*

A Mann–Whitney *U* test was conducted at 0.05 significance level, to show any significant difference of expansion between directions.

### 2.12 4D printing

Fig. S9 in ESI,† shows the printing path for the eight [0°, 90°] specimens. After printing, the hydrogels were cross-linked and then positioned on a polytetrafluoroethylene (PTFE) film to dry. Side-view photos were taken with Allied Vision 1800 U–234 m after 24 hours when the specimens stopped moving. These photos were imported in Matlab. Eight points were positioned on the photo along the length of the specimens. Finally, the curvature of the specimen was measured by interpolating these points. Fig. S10 in ESI,† shows the printing path as well as the geometry of the print used for the part designed to demonstrate the 4D printing capabilities. The cross-linked rosette was then positioned on a PTFE sheet to avoid abrasion. The part was left in a controlled environment room at 23 °C and 42% relative humidity. The actuation was recorded with an EPL zoom webcam 2 MPixel camera.

## 3 Results and discussion

### 3.1 Single fibres test

**Fig. 3:**
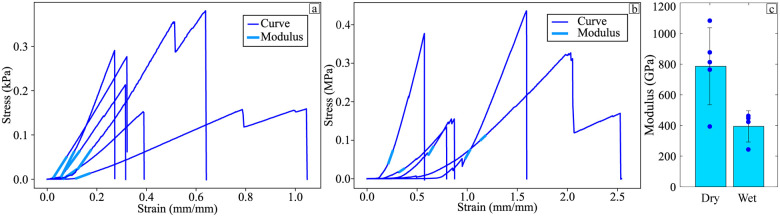
Stress–strain curves for the single fibre testing tests conducted with (a) dry fibres (b) wet fibres. (c) Distribution of Young's modulus.

A single fibre test was used to determine, the impact of the alginate-poloxamer hydrogel on the flax fibres. It was also used to gain a better understanding of the role of the fibres in the mechanics of the hydrogel composites. The stress–strain curves used to obtain the modulus of the wet and dried flax fibres are presented in Fig. 3(a) and (b), respectively. Five values for the dried specimens and four for the wet specimens were considered by removing outliers until the coefficient of variation became lower than 30%. The resulting moduli were 394.1 ± 88.2 MPa and 786.1 ± 224.6 MPa as shown in Fig. 3(c). The humidity conditioning halved the stiffness, giving a knockdown ratio *ψ* of 50.1%. This showed that the hydrogel as a surrounding matrix had a significant negative impact on the properties of the overall composite. The flax fibre has the potential to be even more efficient when considering other hydrogel compositions.

#### 3.1.1 Dimensions of the fibre after milling

**Fig. 4:**
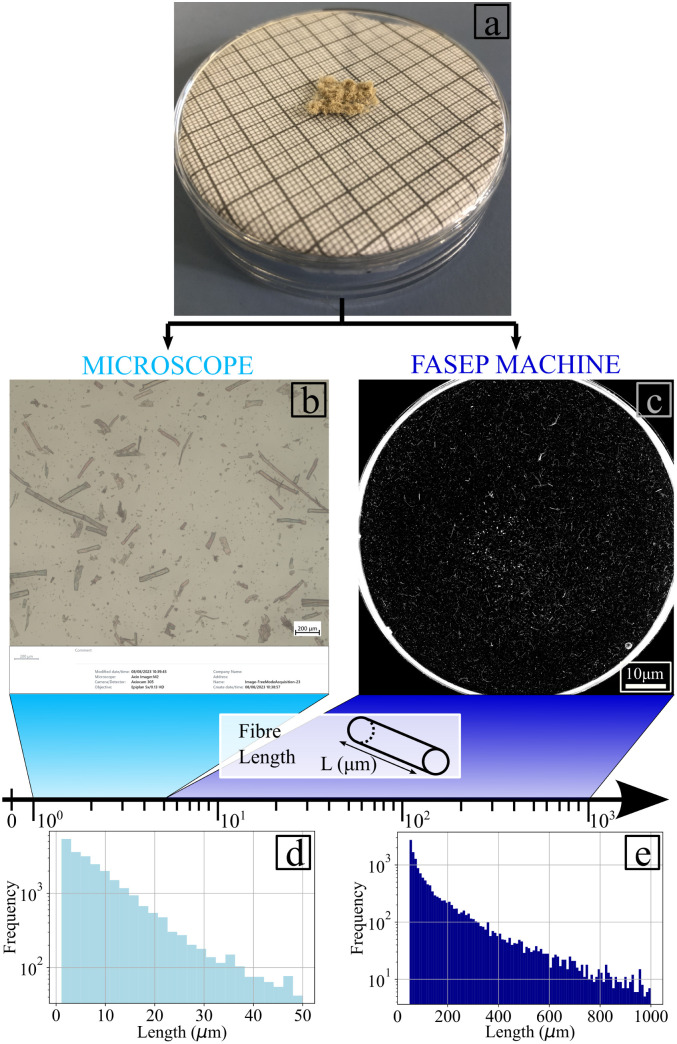
Procedure to measure the properties of the flax fibres. (a) raw milled fibres. (b) Example of microscope image taken to measure the dimension of the fibres. The length here ranges between 1 and 50 μm. (c) Example of image taken by the IDM system FASEP machine to obtain the dimensions of the fibres if their length ranges between 50 μm and 300 μm. (d) Histogram of the distribution of the fibres lengths measured with the optical microscope. (e) Histogram of the distribution of the lengths for fibres dimensions measured with the FASEP machine.

The technique used to mill the flax fibres (see Fig. 4(a)) created a distribution in the size of the reinforcements. Fig. 4 presents the images (Fig. 4(b) and (c)) and the flax fibre length distributions (Fig. 4(d) and (e)) obtained during the test.

**Fig. 5:**
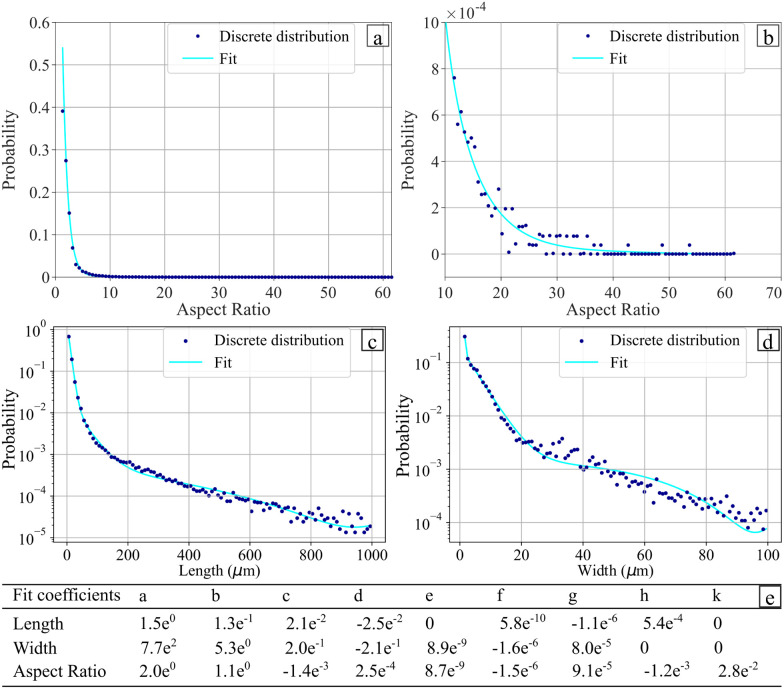
Distribution and interpolation of: (a) length and (b) width. Distribution and interpolation of aspect ratio (c) large view (d) zoomed in view. (e) Interpolation coefficients for the fit function of the different dimensions.

The distribution of the fibres length and width are presented in Fig. 5(c) and (d), respectively. Fig. 5 shows that 90% of the fibres are for the most part below 25 μm in length and 15 μm in width. The great majority of the fibres (95% of them) have aspect ratios between 1 and 5 (Fig. 5(c) and (d)) and can be therefore classified as being powder-like. The Cox–Krenchel equation shows that the longer the fibres, the stiffer the reinforced composite.^60,61^ Further increase in terms of stiffness could also be achieved by improving the ball mill process. Table in Fig. 5(e) indicates the coefficients of the interpolation functions of the three structural properties evaluated. The coefficients of correlation are equal to 0.991, 0.998 and 09975 for the length, width and aspect ratio, respectively. The genetic algorithm used to obtain a fibre size distribution representative of the material was implemented with a population size of 2200, population deleted each round of 1800, best population going to the next round of 100 and the number of crossovers per round equal to 100. The maximum correlation coefficient obtained *via* the algorithm was 0.97.

### 3.2 Select fibre weight fraction

#### 3.2.1 Printing

**Fig. 6:**
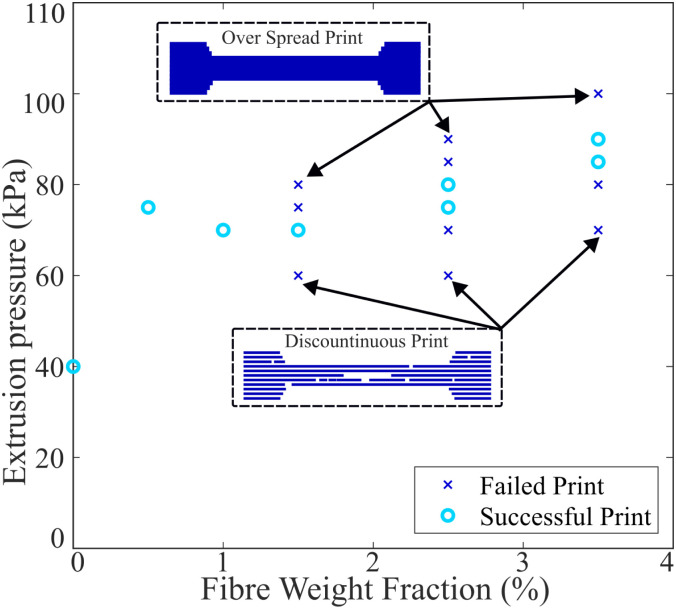
Successful and failed prints for the different fibre weight fractions used to produce the hydrogel composites. The pressure was measured on the printer's monitoring display. The schematics present the different issues faced with inappropriate printing pressure. For too low pressure the print is discontinuous. On the other hand, too high pressure leads to over spread of the material and not precise geometry.

Fig. 6 shows the different testing parameters used to print the alginate-poloxamer hydrogels with different weight fractions of flax fibre. The failed prints involved insufficient extrusion of material, or discontinuous extrusion. The unsuccessful extrusions also involved prints in which too much material was printed by not respecting the dimension assigned to the G-code.

The figure also indicates the pressure required for printing various levels of reinforcement within the hydrogel. At a given printing speed, a minimum extrusion pressure is necessary to enable the printing of the material. Typically, this minimum pressure increases with the addition of larger amounts of reinforcement. There was, however, limited flexibility to adjust the printing parameters when the fibre weight fraction exceeded 1.5%. It should be noted that no prints with a fibre weight fraction exceeding 3.5% were successful.

**Fig. 7:**
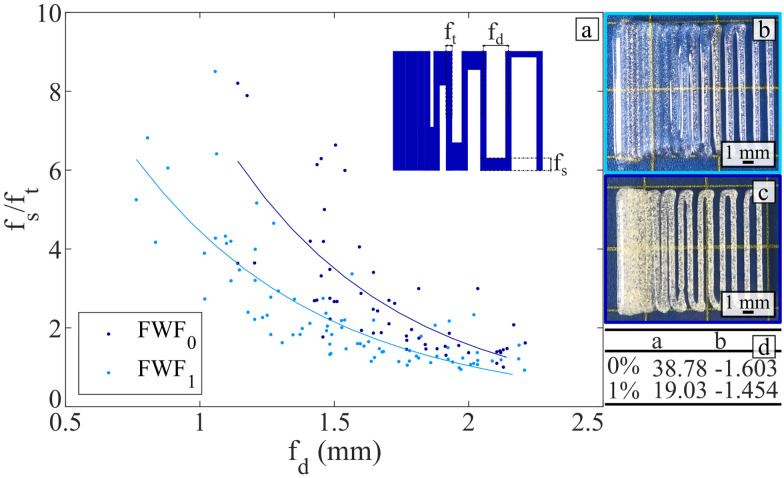
Filament fusion test results. (a) Geometric parameters for fusion of the printed filament. The schematic on the top right present the different parameters displayed. An exponential interpolation (*f*: *x* → *a* × *e*^*b*×*x*^) is provided on the graph. (b) Example of printed specimen for a fibre weight fraction of 0%. (c) Example of printed specimen for a fibre weight fraction of 1%. (d) Coefficients obtained for the exponential interpolation displayed in (a).

The fusion and resolution of the printings were tested, and the results are presented in Fig. 7. Fig. 7(a) demonstrates that the addition of flax fibres enables printing with a closer distance between filaments without them fusing. One of the reasons for this is the higher resolution achieved for the printed filament when incorporating flax fibres during printing. There is a statistically significant difference (Mann–Whitney test: *p* = 0.0002) of 8% between the thickness of the filament printed with and without flax fibres. Therefore, adding flax fibre permits lowering the minimum pore size.

**Fig. 8:**
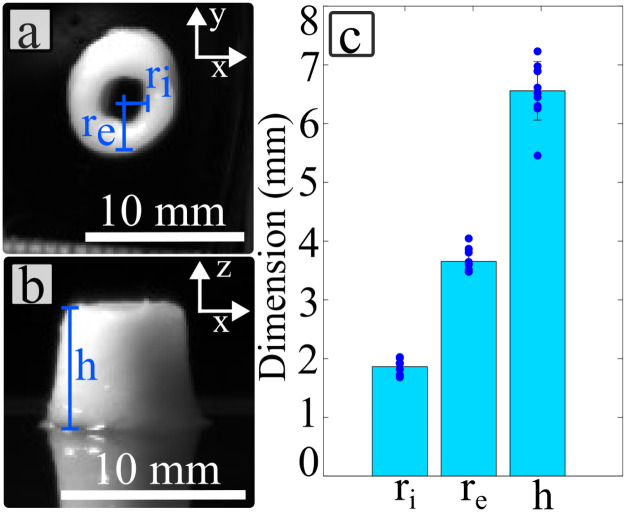
Annulus scaffold test specimens. (a) Top view of a annulus scaffold. (b) Side view of an annulus scaffold. (c) Statistical distribution of the dimension of the annulus scaffold.

The improved resolution and stability of the printed filament, achieved through the addition of flax fibre, enabled the production of the small annulus scaffold shown in Fig. 8. The coefficient of variation obtained for the three dimensions displayed in Fig. 8 are below 10% (6.8%, 5.3% and 7.6% for *r*_*i*_, *r*_e_ and *h*, respectively). This consistent repeatability in dimensions demonstrates the material's ability to produce annulus-shaped structures reliably. The addition of flax fibres allowed to reach heights of 6.55 ± 0.49 mm, which are clinically-relevant dimensions for annulus scaffolds.^62–64^

#### 3.2.2 Tensile test

The alginate-poloxamer hydrogels with different flax fibre weight fractions that were successfully printed were compared by tensile testing. An example of the curves obtained for a given fibre weight fraction is given in Fig. S2 (ESI†), which was used to determine the two stiffness *E*_1_, *E*_2_ the strength *σ* and the strain at failure *ε*.

**Fig. 9:**
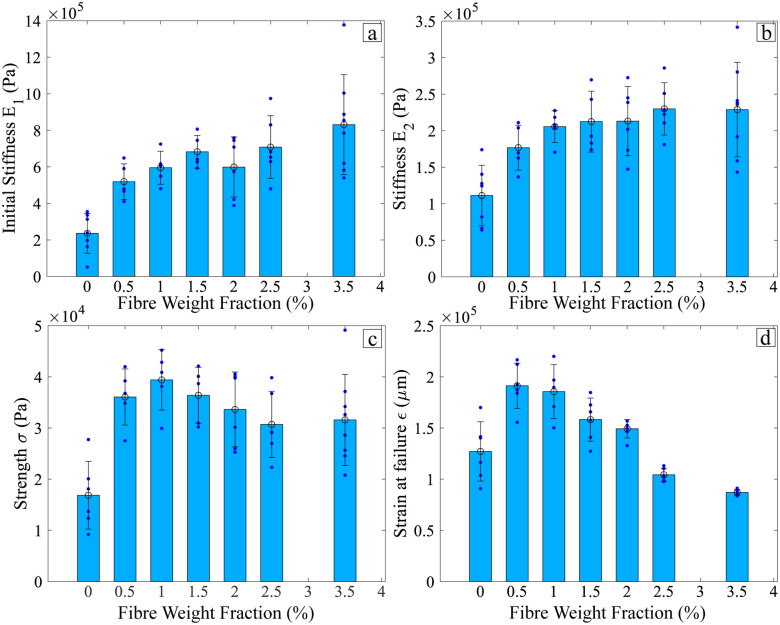
Influence of the flax fibres reinforcement on the mechanical properties of the alginate-poloxamer hydrogel. (a) *E*_1_: initial stiffness [0; 10 000] *με* (b) *E*_2_ stiffness [50 000; 70 000] *με* (c) *σ* strength (d) *ε* strain at failure.

Fig. 9 shows the influence of the flax fibre weight fractions on the mechanical properties of the alginate-poloxamer hydrogel. Fig. 9(a) and (b) show that the hydrogel is stiffer with flax fibre reinforcement. The KrusKal Wallis tests were run to show that the two moduli, *E*_1_ (*p* = 1.0) and *E*_2_ (*p* = 1.0), are almost identical for flax fibre weight fractions between 1.0% and 3.5%. However, within this range, the standard deviation also increased with higher fibre weight fraction, resulting in lower repeatability of the mechanical properties of the composites. In addition to the interdependence with the amount of flax fibre, the stiffness of the composite is also dependent upon the amount of poloxamer and alginate. Xu *et al.* have shown that different classes of poloxamers (P188 and P407) in alginate hydrogels cross-linked with calcium chloride lead to different compressive stiffness and indentation resistance at constant alginate content, although high concentrations of P407 hindered the cross-linking and the compressive stiffness.^65^ Popescu *et al.* show that the increase of the weight fraction of alginate in a poloxamer hydrogel lowers the compression strength and stiffness of the material.^66^ In all those references, the poloxamer's impact on the hydrogel's stiffness was also described as dependent on the amount of alginate used in the production. The materials of this work showed stiffness levels similar to those found in the majority of the available open literature (see Fig. 1).

Fig. 9(c) shows the influence of flax fibre weight fraction on the strength of the alginate-poloxamer hydrogel. The composite was at its strongest for a fibre weight fraction of 1%; this makes this composition the most suitable for load-bearing applications. Fig. 9(d) displays the influence of reinforcement on the strain at failure of the composite. In this case, the largest strain at failure was for fibre weight fractions of 0.5% and 1.0%.

**Fig. 10:**
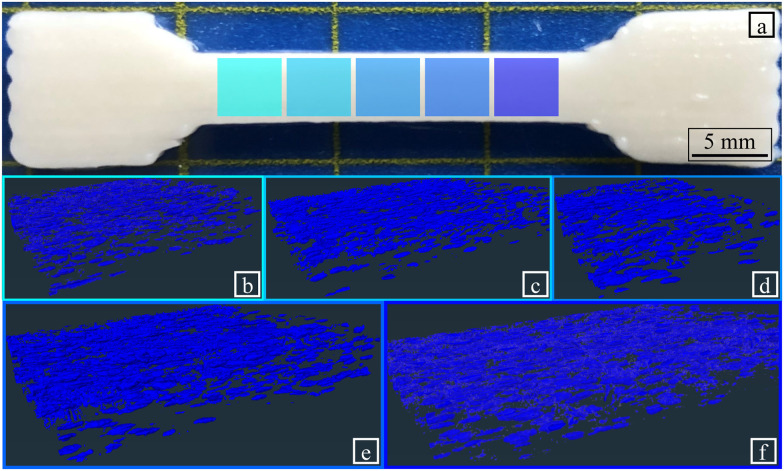
Post-processing of the images acquired *via* CT-scanning. Each grade of blue presents one section considered for measuring the porosity. (a) Specimen considered for CT-scanning. (b) Section 1, (c) Section 2, (d) Section 3, (e) Section 4, (f) Section 5 visualisation of the porosity in the different sections of the specimen presented in (a). The overall porosity measured in the specimen is 6.9 ± 1.1%.

A computed tomography (CT) scan performed on specimens before tensile testing, revealed the presence of microscale voids of varying shapes and sizes, as shown in Fig. 10. Not accounting for pores smaller than 6 μm, the microscale porosity was found to be 6.9 ± 1.1%. Slightly higher porosity was observed on the edges of the specimen than at the centre, with a reasonably homogeneous internal geometry observed inside the specimens.

**Fig. 11:**
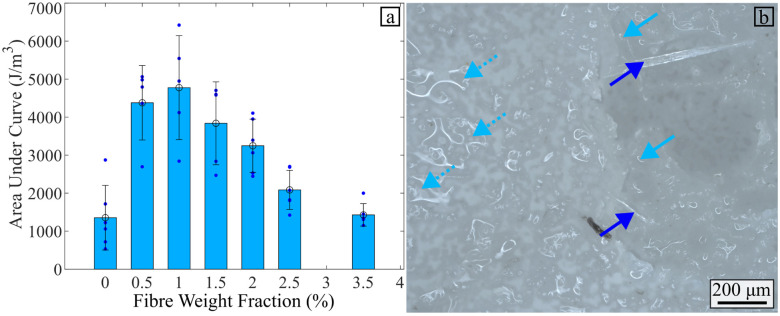
(a) Influence of the amount of flax fibre on the area under the curve (AUC) measured. (b) Optical microscope image of failed specimen cross-section. Dark blue arrows pointing upwards show the fibres pull out during fracture. Dash light blue arrows pointing downwards indicate large voids on the surface of the specimen. Light blue arrows pointing downwards show the large voids split during fracture.

Fig. 11 shows the influence of the flax fibre content on the energy dissipated during the fracture of the composite. The figure also presents an image of a cross-section of a failed specimen to provide more insight into the fracture process within the composite hydrogel.

**Fig. 12:**
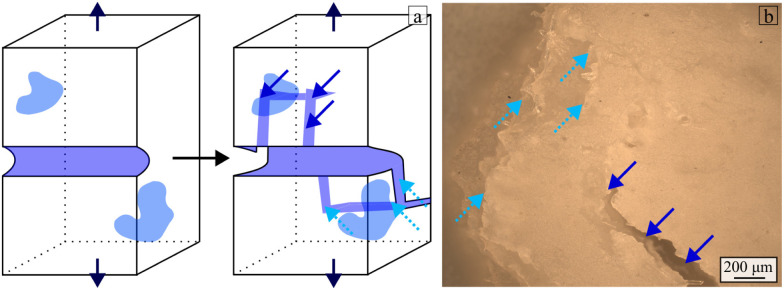
Crack branching mechanism. A main crack propagates through the material and branches when it encounters defects such as large voids or fibres in the vicinity of its path. The light blue arrow pointing upwards describes the main crack running through the specimen. The dark blue arrows pointing downwards indicate a non-catastrophic crack (called branch) from the main fracture crack. (a) Schematic. (b) Microscope Image.

The dark blue arrows in Fig. 11(b) show fibres being pulled out of the hydrogel during failure. The fibre pullout is a source of energy dissipation that partially explains the increase in energy dissipated when the fibre weight fraction increases from 0% to 1%. The light blue arrows pointing downwards indicate the presence of large voids broken during the tensile tests. The fibres and the voids highlighted in Fig. 10 are sources of defects in the hydrogel composite and lead to a deflection of the crack path. The crack jumps from one large void or fibre to another, thus creating discontinuities in the material. The full light blue arrows in Fig. 11 present examples of the surfaces formed when the crack jumps from one propagation plane to the other.^67^ These crack jumps provide insight into a potential crack branching mechanism. Crack branching sites were indeed observed in the specimens, as observed in Fig. 12. A schematic of the crack-splitting mechanism is presented in Fig. 12(a). The sites of the crack splitting provide evidence that several cracks propagate in parallel before the failure crack propagates through the section of the composite. For instance, Fig. 12(b) shows a crack that failed one of the hydrogel composite samples. The dark blue arrows on the same image indicate a crack (branch) that propagated in parallel to the main crack. A larger fibre weight fraction increases the chance of branches appearing, therefore leading to more material failure and more energy dissipation. As shown in Fig. 9(d), the strain at failure decreases for flax fibre weight fractions above 1%. This leads to less material stretch during failure and, consequently, less energy being dissipated during the deformation of the material. This partly explains why the energy dissipated decreases when the flax fibre weight fraction exceeds 1%.

The coefficients of variation of all the mechanical properties measured are presented in 13. This variability is partly attributed to the manual gripping during the loading process of the specimens, which are, reasonably fairly fragile and could have deteriorated when positioned in the grips. The humidity and temperature during the preparation of the hydrogel could also affect the quality of the printed structure. The numerous tests performed involved a test campaign lasting several weeks; the variations in temperature and relative humidity during this period led to the variability in the results. The average coefficient of variation observed on 51 data points from open literature data (Fig. 1) is 34.3%. The variability observed for the hydrogel composites of this work is within the same order of magnitude compared to the one observed in the previous studies described in the literature.

The annulus scaffold constructs printed and displayed in Fig. 8 were loaded in compression; the stress–strain curves obtained up to a 8 Newton load could be observed in Fig. S5 (ESI†). The modulus was 869 ± 242 kPa. These values are structural and not material properties. Therefore, it is compared in terms of magnitude with human tissues, exhibiting a stiffness order of magnitude similar to that of skin, muscle and cartilage.^68,69^

#### 3.2.3 Hyperelastic properties

The Mooney Rivlin coefficients for describing the tensile properties of the hydrogels are presented in Table 3. For the hydrogel without fibres, the coefficients of variation are 21.4% and 28.4% for the *C*10 and *C*01 coefficients, respectively. For the fibre reinforced hydrogels they are 15.6% and 11.7%, respectively. Sources of variability similar to those described for Table 2 can be used to explain the values in Table 3. The Moonley Rivlin model interpolates the stresses inside the alginate-poloxamer hydrogels over the full range of strain tested (see Fig. 9). The Young's moduli presented in Fig. S2 (ESI†) and in open literature only characterises the mechanical behaviour of the hydrogel within a small strain range. The hyperelastic models therefore cover a broader spectrum strain and could be used for modelling and design aspects.

**Table 2:** Coefficient of variation of mechanical properties: *E*_1_: Young's modulus [0; 10 000] *με*, *E*_2_: Young's modulus [50 000; 70 000] *με*, *σ*: strength, *ε*: strain at fracture, AUC: area under the curve, FWF: fibre weight fraction

FWF	0%	0.5%	1%	1.5%	2%	2.5%	3.5%
*E*_1_	45.9%	18.8%	15.2%	13.1%	27.5%	24.3%	33.0%
*E*_2_	37.3%	17.3%	10.6%	19.6%	22.3%	15.6%	28.3%
*σ*	39.3%	15.2%	15.0%	15.0%	21.7%	21.0%	28.2%
*ε*	22.8%	11.5%	14.2%	13.3%	13.3%	6.1%	5.9%
AUC	62.9%	22.4%	28.7%	28.4%	28.4%	21.5%	24.7%

**Table 3:** Mooney Rivlin coefficient for hydrogel with and without fibres

	*D*1	*C*10 (Pa)	*C*01 (Pa)
Without fibre	0	−7.43 × 10^4^ ± 1.59 × 10^4^	1.22 × 10^5^ ± 3.45 × 10^4^
With fibre	0	−2.06 × 10^5^ ± 3.22 × 10^4^	2.88 × 10^5^ ± 3.38 × 10^4^

### 3.3 Collapse test

**Fig. 13:**
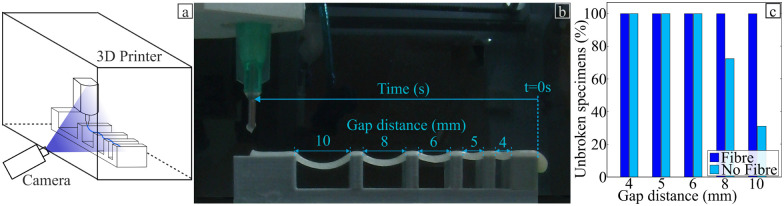
Collapse test experiment. (a) Schematic of the test. (b) Photo of the experiment with the distance between two support block. (c) Collapse test, percentage of unbroken hydrogel for the different distances tested in the collapse test.

Based on the results of the mechanical testing, only the hydrogel with 1.0% fibre weight fraction of flax fibre was considered in the rest of the study. To optimise 4D printing applications, handling materials that can withstand large deformations and possess a higher strain-to-failure capacity is crucial. A common collapse test shown in Fig. 13 was used to observe the impact of the flax fibres on the printing of the alginate-poloxamer hydrogel. Fig. 13(c) shows the percentage of unbroken hydrogels for the different distances evaluated. The hydrogel remained intact for distances of 4 mm, 5 mm, and 6 mm, regardless of whether or not the hydrogel was reinforced with flax fibres. However, the non-reinforced hydrogels failed 30% and 70% of the time for distances of 8 mm and 10 mm, respectively, which could be prevented by reinforcing the hydrogel with flax fibres. Thus, the addition of flax fibres appears to constitute an efficient way of improving the-self suspendable ability of the hydrogel, particularly for designs containing unsupported regions such as the one presented in Fig. S10(a) (ESI†).

### 3.4 Rheology

**Fig. 14:**
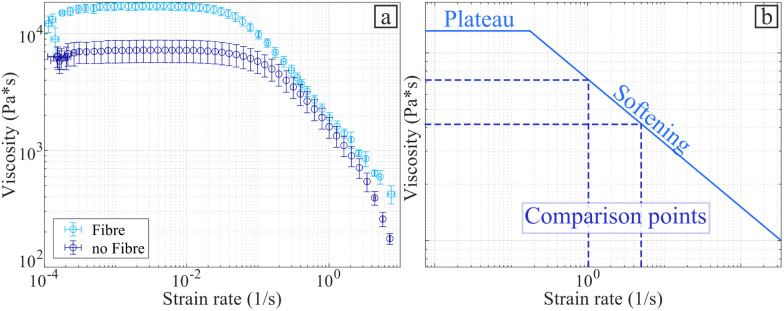
Viscosity with and without the flax fibres. (a) Strain rate *versus* complex viscosity for fibre and without the fibres. (b) Schematic of the viscosity shape.

Fig. S7 in ESI,† shows the stress–strain curve obtained from the rheology test, which was used to determine a shear stiffness is represented by the modulus^70^G* = 71.51 ± 12.33 kPa. The range of validity of this value is between 0 and 1000 *με*. Fig. 14(a) presents the evolution of the viscosity of the hydrogel by varying the strain rate.

Fig. 14(a) shows that the flax fibres increase the viscosity of the hydrogel within the range of strain rates here considered. Fig. 14(b) presents the general shape of the viscosity *versus* the strain rate for the alginate-poloxamer hydrogel reinforced with 1.0% fibre weight fraction of flax fibres, compared to an unreinforced hydrogel. The curve is made of a plateau followed by a softening part for higher strain rates. An analysis of the existing literature related to rheometry and viscosity of 3D printed alginate hydrogels is shown in Table 4. Two types of uncertainty related to the effect of viscosity in hydrogels for bioprinting can be encountered in the open literature. First, the printing path used to produce the specimen is often not reported and often only one specimen is used and evaluated. The only common interval of strain rate values in the literature shear viscosity tests was between 1 s^−1^ and 5 s^−1^. Thus these values were used to assess the stiffening coefficient provided by the flax fibre reinforcements. The addition of flax fibre increased the viscosity of the 3D printed alginate-based hydrogel by 19.5% and 129.2% at strain rates of 1 s^−1^ and 5 s^−1^, respectively. These stiffening coefficients (SC) are presented in Table 4.

**Table 4:** Stiffening coefficient (SC) found in literature. Values adjusted to the weight percentage of reinforcement

Paper	Reinforcement	SC at 10^0^ (%/%)	SC at 5 × 10^0^ (%/%)
This study	Flax	19.5	129.2
71	Simvastatin	19.2	22.1
72	Tri calcium sillicate	[−7.5; 85.7]	[−7.6; 57.9]
73	LAPONITE®	[135.6; 138.9]	[161.8; 162.1]
74	Nanoclay	[7.7; 205.9]	[9.2; 139.1]
75	LAPONITE®	[632.14; 16650]	[232.14; 3852.38]
12	Curcumin	0.0	0.0
76	Carrageenan	[23.33; 74.07]	[122.22; 151.85]
77	Cellulose nanofibres	[318.18; 1254.55]	[318.18; 1254.55]
78	Cellulose nanofibres	−2.53	−2.89
79	Cellulose nanofibres	[−23.33; 37.5]	[−10.71; 46.43]
80	Cellulose nanofibres	[24.64; 600]	[17.19; 3585.86]

Nanocellulose fibres have shown their potential in stiffening hydrogels by adding up to 10^3^% per weight percentage addition. Cellulose have also shown softening potential for certain types of hydrogels and fibres. The flax fibres are mostly made of cellulose and hence, similar chemical interactions are present with the hydrogel.^81^ The main difference with the cellulose used in other works is the scale of the fibre dimensions; those studies use nanofibers while the flax fibres used here are in the range of micrometres. Another class of reinforcement materials are particles such as algae, nanoclay and LAPONITE®. A similar range of stiffness change can be also achieved by using these particles, up to 10^3^%. The flax fibres appear to stiffen the material in shear with the same order of magnitude of other materials used to reinforce hydrogels, except for the case of the nanocellulose and LAPONITE® reinforcements.

### 3.5 4D printing

The density measured for the hydrogel with and without reinforcement is presented in Table 5.

**Table 5:** Density (g cm^−3^). (FWF: fibre weight fraction)

	Wet	Dry
FWF 0%	1.28 ± 0.04	1.53 ± 0.04
FWF 1%	1.28 ± 0.04	1.21 ± 0.06

An effective density of the hydrogel equal to 1.64 g cm^−3^ at 6 μm (the resolution of the CT scanner) was determined *via* the porosity measured through CT scanning. Table 5 shows that the density of the reinforced and non-reinforced wet hydrogel were measured identical. This is unsurprising, given the density of the flax fibres (1.53 g cm^−3 ^^51^) and the alginate-poloxamer hydrogel (1.64 g cm^−3^) are similar. However, the dry flax fibre-reinforced hydrogel were slightly less dense than the non-reinforced hydrogels, as the weight is similar in both specimens and the volume is larger for the reinforced specimens.

The values for the coefficient of moisture expansion (CME) in the different directions of printing were obtained *via* measurement of the mass and volume difference between dry and wet hydrogel. These values are presented in Table 6. The Mann–Whitney showed that no statistical difference was observed between the CME along the width and the thickness directions. For the two fibre weight fractions (1% and 0%) the CME are described as statistically identical (*p* = 0.095) and (*p* = 0.15), respectively. Consequently, the average of the width and the thickness direction is representative of the transverse direction expansion of the material. This transverse expansion is described in Table 6. The length CME are statistically different between fibre reinforced and not reinforced hydrogel *p* = 0.008. On the other hand, transverse CME are statistically identical between the two types of hydrogel tested *p* = 1.0. Consequently, the 4D printing actuation capability is improved by addition of flax fibre *via* the change in CME in the length direction.

**Table 6:** Coefficient of moisture expansion for the reinforced and not reinforced hydrogel. (FWF: fibre weight fraction)

	Length (*β*_*x*_)	Width (*β*_*y*_)	Thickness (*β*_*z*_)	Transverse (*β*_*yz*_)
FWF 0%	3.22 × 10^−1^ ± 1.75 × 10^−2^	6.26 × 10^−1^ ± 1.31 × 10^−2^	5.73 × 10^−1^ ± 5.31 × 10^−2^	5.99 × 10^−1^ ± 2.71 × 10^−2^
FWF 1%	1.29 × 10^−1^ ± 3.73 × 10^−2^	5.74 × 10^−1^ ± 2.49 × 10^−2^	6.31 × 10^−1^ ± 5.68 × 10^−2^	6.03 × 10^−1^ ± 2.04 × 10^−2^

**Fig. 15:**
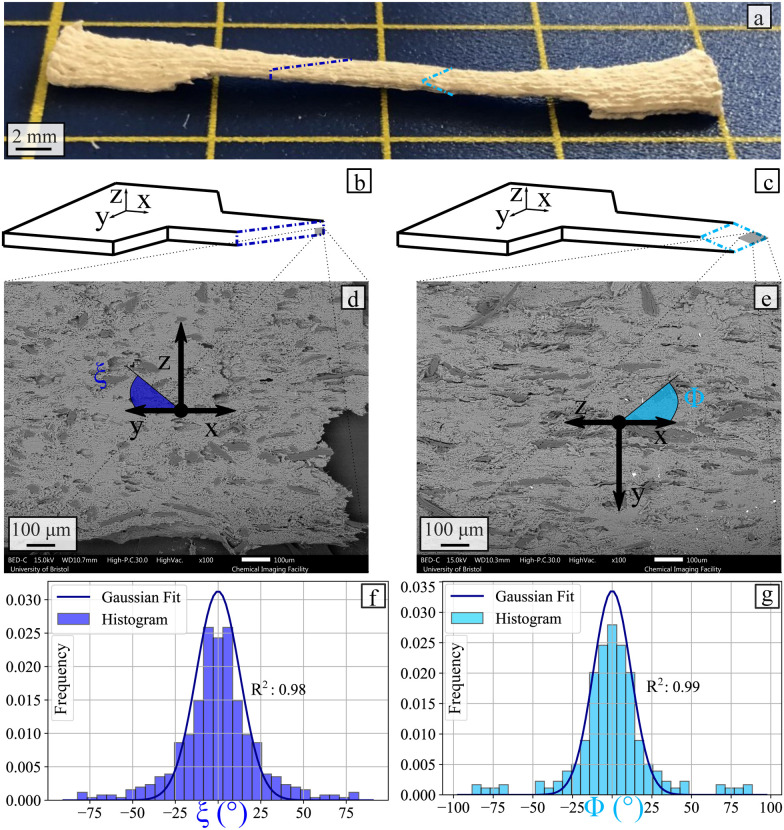
Fibre orientation measurement. (a) Specimen used and cut made in it. (b) and (c) Schematics of the cut made on the specimen. (d) and (e) Example of SEM images perpendicular to the cuts made on the specimen. Fibre orientation. (f) Schematic showing the angles *ξ* and *ϕ* relative to the printing direction (g) distribution of fibre orientation along *ξ* angle (c) distribution of fibre orientation along *ϕ* angle.

The orientation of the flax fibres in the alginate-poloxamer hydrogels was investigated with scanning electron miscroscopy (SEM). A tensile specimen was used for this test as shown in Fig. 15(a). Samples were cut along the width and thickness directions as shown in Fig. 15(b) and (c), then imaged in the cutting plane using scanning electron microscopy, shown in Fig. 15(d) and (e). These micrographs were used to define the orientation of the fibre using two angles (*ξ* and *ϕ*) introduced in Fig. 15(d) and (e). The distributions of the orientation of the fibres observed in the hydrogels are presented in Fig. 15(f) and (g). The measured distributions were both Gaussian-like and centred on 0° due to the symmetric assumption. The standard deviation for the *ξ* and the *ϕ* angles are 12.8 and 11.9, respectively.

As shown with the SEM investigation, the flax fibres are statistically orientated to the printing direction. Therefore, flax fibres limit the contraction of the hydrogel along the printing direction. Hence, as shown with the density presented in 5, the volume of the dry material is larger with the fibres compared to the case without the reinforcements. Table 6 shows the potential for the in-plane deformation of the hydrogel system. The 4D printing potential of a material comes from its ability to expand in different ways along the length and the transverse directions. Without fibre reinforcements, there is a 1.86 : 1 ratio between the transverse and the longitudinal coefficient of moisture expansions. The 4D printing capability of the material is significant due to the statistically different CME in the length and transverse direction (Mann–Whitney test: *p* = 0.008). With fibre reinforcement, this ratio increases to 4.67 : 1. The 4D printing capability of the material is still significant with fibre due to the statistically different CME in the length and transverse direction (Mann–Whitney test: *p* = 0.008). Hence, the presence of the flax fibres appears to modify the 4D printing potential of the alginate-poloxamer hydrogel.

**Fig. 16:**
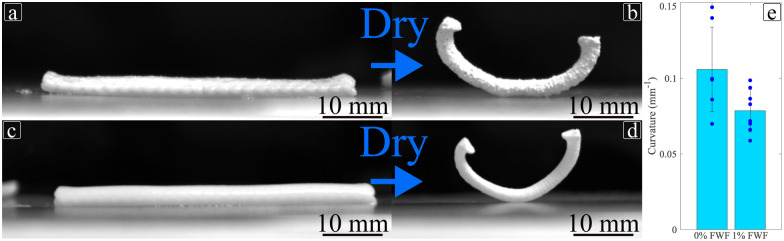
4D printing actuation of [0°; 90°] specimens. (a) and (b) Wet and dry specimen for 1% FWF, respectively. (c) and (d) Wet and dry specimen for 0% FWF, respectively. (e) Comparison of actuation for 1% and 0% FWF.

The difference of CME affects the actuation capability of the material. The difference in stiffness observed in Fig. 9 also impact the ability of the material to actuate.^82^Fig. 16 presents the influence of flax fibre in actuation. The addition of fibres induces a 25.8% lower curvature for a [0°; 90°] stacking sequence. Mann–Whitney test shows a significant statistical difference between the specimens with and without fibres (*p* = 0.0281). The addition of flax fibre stabilises the deformation obtained as the coefficient of variation goes from 32% to 18% when adding the fibres to the hydrogel. Therefore, it is possible to control the mechanical and actuation response to the hydrogel by adding fibres. For instance, Goyal *et al.* produced an alginate-based hydrogel less stiff but more actuation-capable alginate based hydrogel.^83^ For non-alginate-based hydrogel Zheng *et al.* have described the possibility of using them as grip.^84^ Hence, depending on the material to be lifted, the stiffness and actuation capability must be adapted, by changing the amount of reinforcement, the stacking sequence printed.^48,84^

**Fig. 17:**
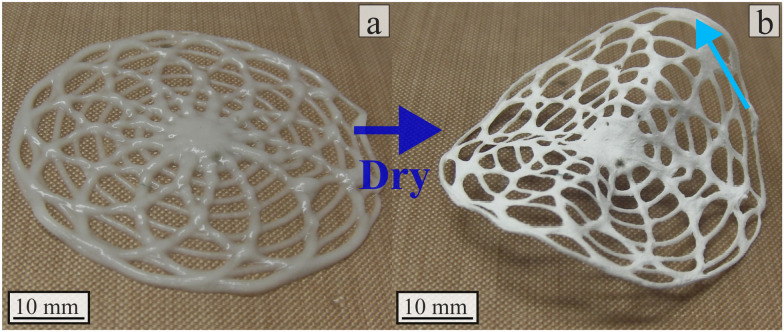
Rosette actuation with 1% flax fibre reinforced alginate-poloxamer hydrogel. (a) Wet specimens in cross-linking material after 24 h (b) dry specimens at room humidity. The blue arrows presents the highest point of the specimen after the actuation. A video in ESI,† shows the full actuation of the structure.

Kirilova *et al.* have shown the possibility of creating tubes from flat 4D printed alginate-based hydrogel.^85^ Further exploration of the 4D printing design space is necessary to produce tubes as displayed in Fig. 8 with 4D printed technique. One potential exploration axis is to get inspired by existing geometries in nature.^49,84^Fig. 17 presents two examples of the 4D printing using room humidity actuation. Rosette structures (inspired like Goyal *et al.* by flowers^83^) were printed using alginate-poloxamer hydrogel reinforced with 1% flax fibre weight fraction. Upon drying the specimen went from flat to 8 mm high on its highest point. This test shows the 4D printing actuation potential of this material for smart material applications.

## 4 Conclusion

This work provides insight into the production and performance of a new composite biomaterial made from flax fibres reinforcing an alginate-poloxamer hydrogel. This material is intended for potential structural and 4D printing applications using biobased components. The work provides information and data to model such material *via* characterisation of the internal microstructure. Then single fibre tests showed that alginate-poloxamer hydrogel conditioning decreased the stiffness of the flax fibres by 50.1%. After milling the flax fibres used to reinforce the hydrogel, their dimensions were measured. After adding the fibres to the alginate-poloxamer hydrogel, optimised printing configurations for the hydrogel were identified. Collapse tests showed the ability of flax fibres to improve the self-suspendable capability of the hydrogel. The fusion filament tests also showed improved resolution *via* the addition of flax fibres, but also the potential to print smaller pores. Mechanical tensile tests showed that a 1% flax fibre weight fraction provided the optimal values of strength, strain at failure, and energy dissipated during fracture. The stiffness of the 1% flax fibre weight fraction was shown to be statistically identical to composites with larger weight fibre fractions, but statistically more repeatable. The fracture analysis of the composite highlighted the benefit of adding flax fibres to improve the toughness of the material. The 1% fibre weight fraction of reinforcement was used in the remainder of the study. Parameters to model the material were identified from tests and compared against open literature. The hydrogel mechanical properties were described with the two parameters Mooney-Rivlin models (*C*10 = 394.1 Pa; *C*01 = 207.4 Pa). The embedding of flax fibres improved the ability of the alginate-poloxamer hydrogel to be printed without support over long distances. Rheology tests showed that the flax fibres provided significant increases in terms of viscosity compared to the baseline hydrogel, with shear stiffness augmented to 39% and 129% at the strain rates comparison points of, 1 rad s^−1^ and 5 rad s^−1^, respectively. The dispersion of the flax fibres also allows to control 4D printing actuation of the composite hydrogel, as it changes the difference in the coefficient of moisture expansion between the axial 1.29 × 10^−1^ and transverse 6.03 × 10^−1^ directions. Adding the flax fibres lowered the actuation curvature 25% but improved the repeatability of the actuation obtained. The coefficient of variation for the final curvature measured was reduced by 43% after adding the fibres. The comparison between rheology and tensile properties of other hydrogel systems in open literature clearly shows the potential of flax fibres to be used as structural reinforcement in these hydrogel materials and attractive 4D printing actuation capabilities under different humidity conditions.

## Author contributions

Charles de Kergariou: funding acquisition, investigation; methodology; data curation; writing – original draft; conceptualisation; formal analysis, supervision, project administration, resources; Graham J. Day: resources; Adam Perriman: supervision; James Armstrong: supervision; writing – review & editing; Fabrizio Scarpa: supervision, methodology, writing – review editing, conceptualisation, funding acquisition, project administration, resources.

## Conflicts of interest

There are no conflicts to declare.

## Supplementary Material

SM-020-D4SM00135D-s001

SM-020-D4SM00135D-s002
