# Correction: Flax fibre reinforced alginate poloxamer hydrogel: assessment of mechanical and 4D printing potential

**DOI:** 10.1039/d4sm90089h

**Published:** 2024-06-06

**Authors:** Charles de Kergariou, Graham J. Day, Adam W. Perriman, James P. K. Armstrong, Fabrizio Scarpa

**Affiliations:** a Bristol Composites Institute, School of Civil, Aerospace and Design Engineering (CADE), University of Bristol, University Walk Bristol BS8 1TR UK hl18503@bristol.ac.uk charles.dekergariou@bristol.ac.uk; b Biomedical Engineering, James Watt School of Engineering, University of Glasgow Glasgow UK; c School of Cellular and Molecular Medicine, University of Bristol BS8 1TD Bristol UK; d Research School of Chemistry and John Curtin School of Medical Research, Australian National University Canberra ACT2601 Australia; e Department of Translational Health Sciences, Bristol Medical School, University of Bristol BS1 3NY Bristol UK

## Abstract

Correction for ‘Flax fibre reinforced alginate poloxamer hydrogel: assessment of mechanical and 4D printing potential’ by Charles de Kergariou *et al.*, *Soft Matter*, 2024, **20**, 4021–4034, https://doi.org/10.1039/D4SM00135D.

The authors regret that incorrect versions of eqn (4) and (5) were included in the original article. The correct versions of the equations are shown below.

 

Eqn (4):

*ρ* = *m*/(*l* × *t* × *w*)

 

Eqn (5):

CME = ((*d*_wet_ − *d*_dry_)/*d*_dry_ × 100)/((*m*_wet_ − *m*_dry_)/*m*_dry_ × 100)

 

The Royal Society of Chemistry apologises for these errors and any consequent inconvenience to authors and readers.

