# Supplementary material for: Flax fibre reinforced alginate poloxamer hydrogel: assessment of mechanical and 4D printing potential
Source: Soft Matter. 2024 May 2;20(19):4021–34. doi: 10.1039/d4sm00135d (PMC11095501; doi:10.1039/d4sm00135d)
Supplement: SM-020-D4SM00135D-s001 [file SM-020-D4SM00135D-s001.pdf]

Electronic Supplementary Material (ESI) for Soft Matter.
This journal is © The Royal Society of Chemistry 2024

Soft Matter

ARTICLE TYPE

Cite this: DOI: 00.0000/xxxxxxxxxx

# ESI: Flax fibre reinforced alginate poloxamer hydrogel: assessment of mechanical and 4D printing potential

Charles de Kergariou*[a], Graham J. Day[bc], Adam W. Perriman[cd], James P.K. Armstrong[e] and Fabrizio Scarpa[a]

Received Date
Accepted Date

DOI: 00.0000/xxxxxxxxxx

This supplementary material presents the printing paths and geometries for the different tests conducted. It also provides a detailled procedure for the production of the composite hydrogels studied. Examples of stress strain curves for the tensile, rheology and annulus scaffold compression tests are also provided.

## 1 Hydrogel production

Tab. S1 The different steps of production of the hydrogel. The ingredients are added from top to bottom. Stock 1 was produced by heating the mixture in the autoclave for 12h. 24h in the fridge was necessary before the mixture to be used. The stock 2 was obtained by mixing the ingredient twice one minute at 3500rpm in a DAQ mixer with manual mixing in between. Finally the printing material was obtained by mixing the material twice for 5 minutes at 3500rpm with manual mixing in between. All these different steps were conducted at room temperature.

| Stock 1 | Stock 2 | Print Material |
|---|---|---|
| Poloxamer 29.95wt% | Stock 1 66wt% | Stock 2 66.7%wt |
| Water 70.05wt% | Sodium Alginate 9wt% | Flax Fibre *n*wt% |
| / | Water 25wt% | Water 23.3-*n*%wt |
| / | / | $CaCl_2$ 10%wt |

[a] Bristol Composites Institute, School of Civil, Aerospace and Design Engineering (CADE), University of Bristol, University Walk, Bristol BS8 1TR, UK; E-mail: hl18503@bristol.ac.uk
[b] Biomedical Engineering, James Watt School of Engineering, University of Glasgow, Glasgow, UK
[c] School of Cellular and Molecular Medicine, University of Bristol, BS8 1TD Bristol, UK
[d] Research School of Chemistry and John Curtin School of Medical Research, Australian National University, Canberra ACT2601, Australia
[e] Department of Translational Health Sciences, Bristol Medical School, University of Bristol, BS1 3NY Bristol, UK
† Electronic Supplementary Information (ESI) available: [details of any supplementary information available should be included here]. See DOI: 10.1039/cXsm00000x/
‡ Additional footnotes to the title and authors can be included *e.g.* 'Present address:' or 'These authors contributed equally to this work' as above using the symbols: ‡, §, and ¶. Please place the appropriate symbol next to the author's name and include a \footnotetext entry in the the correct place in the list.

## 2 Printing path and geometry

### 2.1 Tensile test

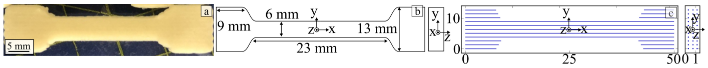


Fig. S1 Tensile test specimens. a): Printed specimens b): Printing dimensions c): Printing path in mm.

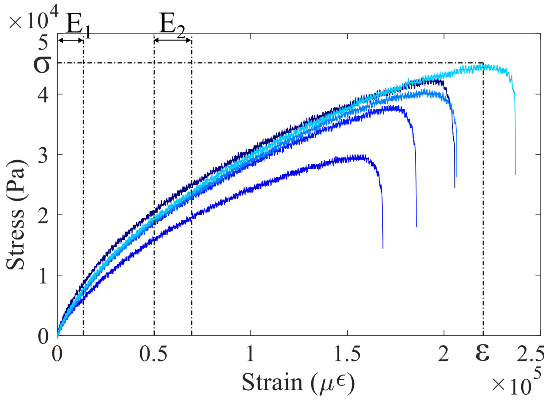


Fig. S2 Stress versus strain curve for alginate-poloxamer hydrogel with 1% flax fibre weight fraction, including definition of the related mechanical properties/engineering constants.

## 2.2 Filament Fusion test

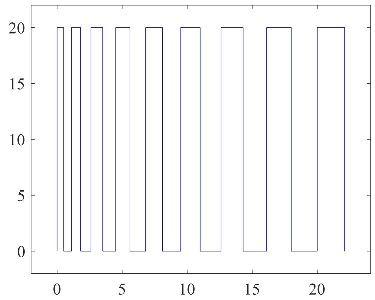

Fig. S3 Printing path for fusion filament test in mm.

## 2.3 Annulus scaffold

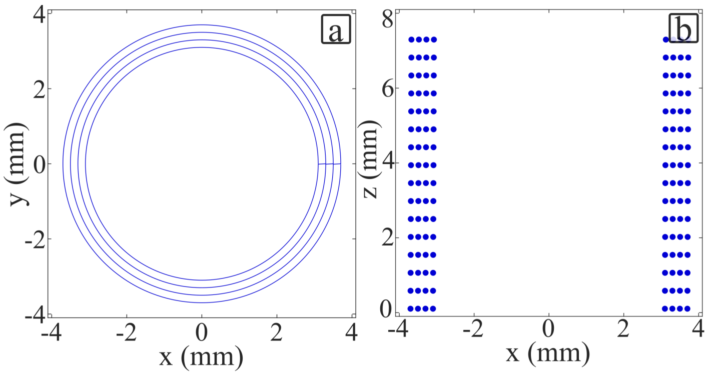

Fig. S4 Printing path to produce cylinder specimen. a): Top view; b): Side view.

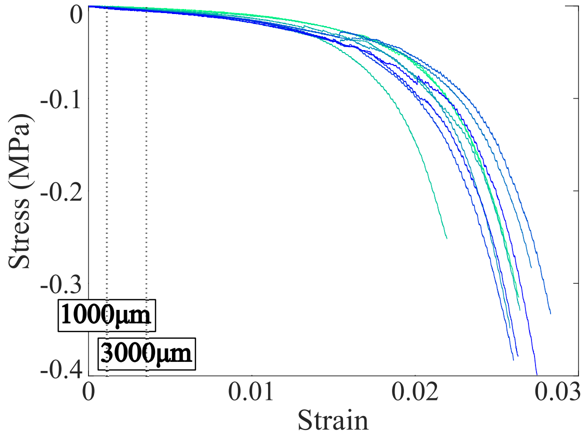

Fig. S5 Stress strain curve for compression test of the cylinder.

## 2.4 Rheology test

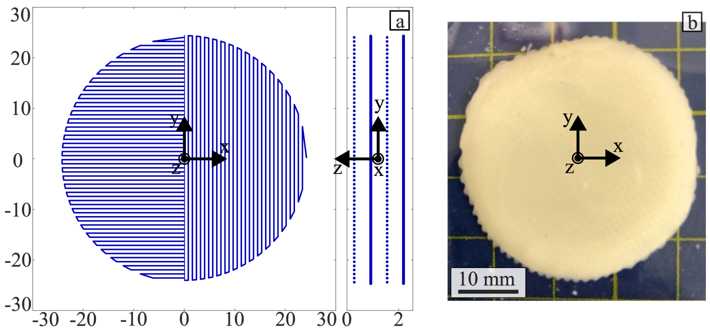


Fig. S6 Rheological specimen. a): Printing path in mm b): Printed specimens.

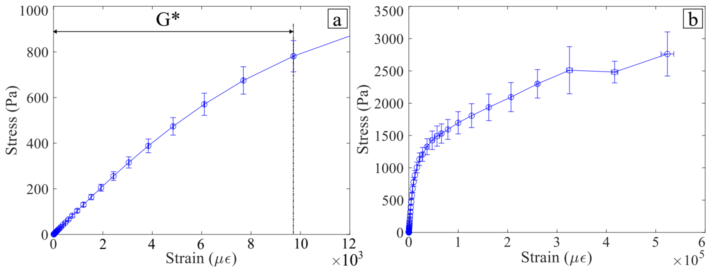


Fig. S7 Stress strain curve rheology for 1% of fibre weight fraction. a): Zoom on the stress range curve used to calculate the complex modulus. b): Full strain range.

## 2.5 Density and Coefficient of Moisture Expansion

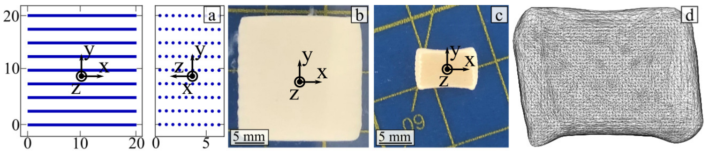


Fig. S8 Density and coefficient of moisture expansion measurement. a): Printing path in mm b): Wet specimen c): Dry specimen d): 3D scanner volume of dried specimen.

## 2.6 4D printing

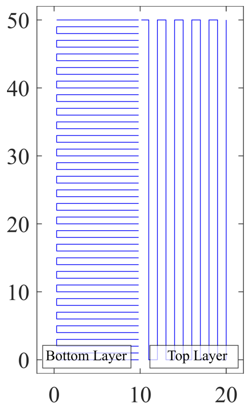


Fig. S9 4D printed path for [0°;90°] specimens in mm.

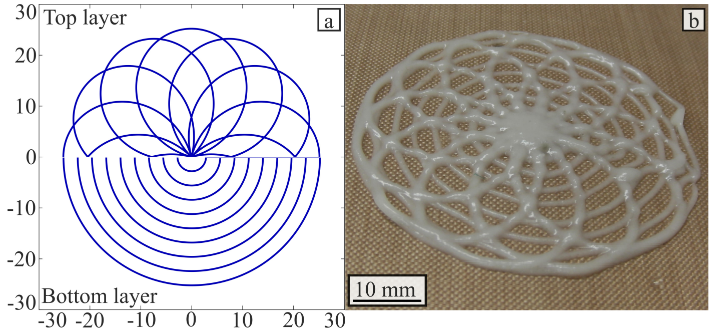


Fig. S10 4D printed rosette. a): Printing path in mm b): Printed and cross-linked rosette.
